# Management of stent-related tracheoesophageal fistula in complex post-tuberculosis tracheobronchial stenosis: A case report

**DOI:** 10.3389/fmed.2022.996140

**Published:** 2022-11-24

**Authors:** Yang Bai, Yuting Yin, Jing Chi, Shuang Li, Yishi Li, Shuliang Guo

**Affiliations:** ^1^Department of Respiratory and Critical Care Medicine, The First Affiliated Hospital of Chongqing Medical University, Chongqing, China; ^2^Department of Respiratory and Critical Care Medicine, Chongqing Shapingba District People’s Hospital, Chongqing, China; ^3^Department of Gastrointestinal Surgery, Jinshan Hospital, The First Affiliated Hospital of Chongqing Medical University, Chongqing, China

**Keywords:** post-tuberculosis tracheobronchial stenosis, tracheoesophageal fistula, self-expandable metallic stent, case report, complication

## Abstract

**Background:**

The covered self-expandable metallic stents (SEMS) have been used to manage benign tracheobronchial stenosis, especially the complex post-tuberculosis (TB) tracheobronchial stenosis (PTTS) with cartilage destruction or malacia. This procedure could lead to stent-related tracheoesophageal fistula (TEF).

**Case presentation:**

A 21-year-old woman, who had one covered Y-shaped SEMS inserted to manage complex PTTS 2 years ago, presented with dyspnea and frequent coughing on drinking water. The bronchoscopy confirmed extensive granulation tissue hyperplasia and a TEF on the upper edge of the covered SEMS. The covered SEMS was removed in three steps, and another fully covered Y-shape SEMS (Microtech Co., Ltd., Nanjing, China) was inserted to restore patency in the tracheobronchial tree and occlude the TEF orifice. Recombinant bovine basic fibroblast growth factor (rbFGF) (6,000 IU/time) was sprayed into and around the fistula through the V-System single-use cannula *via* the flexible bronchoscope every other week. The patient showed sustained clinical and radiographic improvement, and the TEF healed.

**Conclusion:**

We presented a three-step bronchoscopic approach to managing a stent-related TEF in a patient with complex PTTS. Subsequently, regular bronchoscopic debridement of granulation tissue developing on the upper edge of SEMS is necessary to maintain the stent patency and reduce the risk of recurrent stent-related TEF. A fully covered SEMS associated with the local administration of rbFGF seems to offer an alternative simplified one-stage procedure for the temporary management of TEF combined with complex PTTS in non-surgical candidates.

## Introduction

Tracheoesophageal fistula (TEF) is a pathological channel between the trachea and esophagus, classified into benign and malignant types (1). The benign TEF usually results from artificial ventilation with the endotracheal tube or tracheostomy tube (2). The covered self-expandable metallic stent (SEMS) has been applied in managing benign tracheobronchial stenosis, especially the complex post-tuberculosis (TB) tracheobronchial stenosis (PTTS) with cartilage destruction or malacia (3–5). Given the accepted use of covered SEMS, it might become an emerging cause for TEF development. If the clinical situation allows, benign TEF could be repaired by surgical treatment, such as select tracheal resection and end-to-end anastomosis, and division and primary repair, of which the success rates were over 94% (6–8). The stent-related TEF in complex PTTS is not frequent and unsuitable for surgical repair. Bronchoscopy has developed from diagnostic to therapeutic methods in treating TEF, as a bridging or alternative procedure to surgery, especially in those unsuitable for surgical repair (9–11). We presented the bronchoscopic management of a stent-related TEF in a patient with complex PTTS.

## Case presentation

A 21-year-old woman, receiving the insertion of one covered Y-shaped SEMS for the treatment of complex PTTS 2 years ago, was admitted to our department with a complaint of dyspnea and frequent choking with water for 2 weeks. The dyspnea was noticed when walking on flat ground or climbing one flight of stairs. She also had sputum, but no fever, night sweats, or weight loss. She had a history of pulmonary TB in 2004, which was cured following the four-drug regimen (2HRZE/10HRE) treatment. The previous bronchoscope had established the diagnosis of complex PTTS with varying degrees of malacia in multiple locations (middle and lower trachea; right and left bronchus), associated with severe obstructive ventilatory impairment. She had repeated respiratory symptoms even after the numerous airway intervention, such as electrocautery, balloon dilatation, and cryotherapy.

**FIGURE 1:**
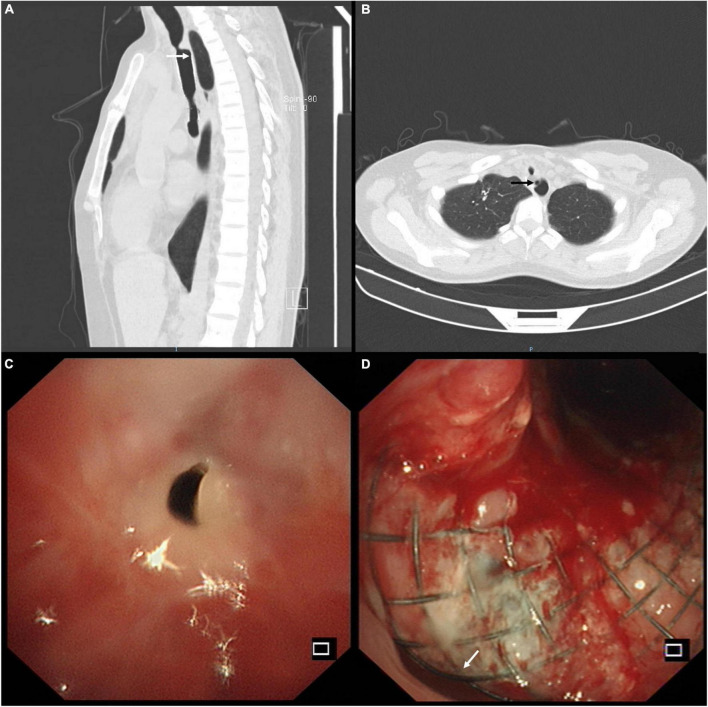
**(A)** Sagittal chest computed tomography (CT) view showed the severe granulation tissue hyperplasia on the upper edge of the covered self-expandable metallic stent (SEMS) and the shearing force against the anterior esophageal wall (white arrow). **(B)** Axial chest CT view showed severe granulation tissue hyperplasia and tracheoesophageal fistula (TEF) (black arrow) on the upper edge of the SEMS. **(C)** The bronchoscopy confirmed severe granulation tissue hyperplasia on the upper edge of the covered SEMS, causing 90% airway obstruction. **(D)** The bronchoscopy identified the TEF (white arrow) located in the 7 o’clock position after eradicating the severe granulation tissue hyperplasia.

She had a temperature of 36.3°C, a heart rate of 112 beats per minute, a breath of 21 times per minute, and a blood pressure of 95/76 mmHg. The physical examination revealed stridor on the anterior neck and rales in the bilateral lower lungs on admission; the rest were insignificant. Laboratory tests showed that leukocyte count was 11.29 × 10^9^/L and neutrophil percentage 81.4%, and the others were unremarkable. The chest computed tomography (CT) demonstrated severe granulation tissue hyperplasia on the upper edge of the covered SEMS, inducing the shearing force against the anterior esophageal wall (Figure 1A), and a part of the stent penetrated the esophagus (Figure 1B). The bronchoscopy confirmed severe granulation tissue hyperplasia on the upper edge of the covered SEMS, causing 90% airway obstruction (Figure 1C). We made radical incisions toward the granulation tissue with electrocautery, followed by mechanical debulking with the forceps and flexible cryoprobe. After eradicating the granulation tissue, the TEF was located in the 7 o’clock position (Figure 1D).

**FIGURE 2:**
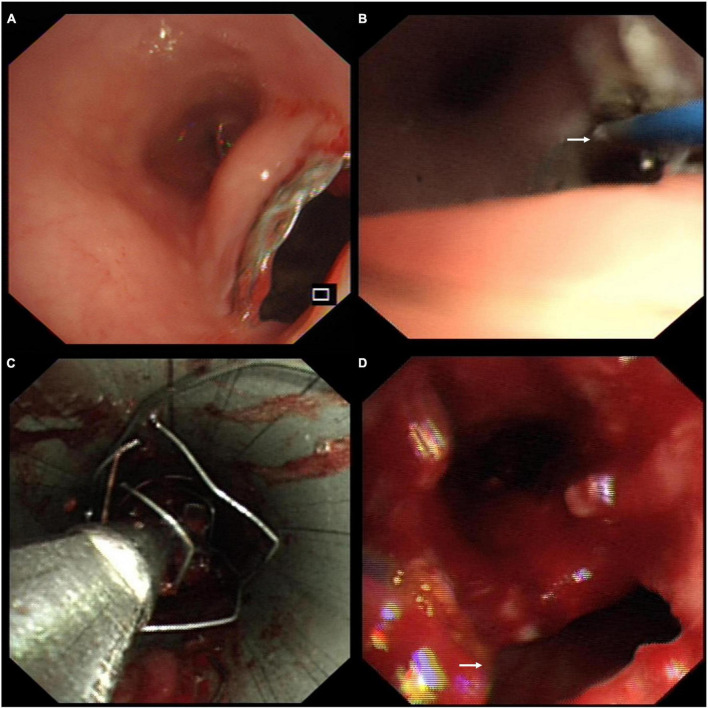
**(A)** Endoscopy showed the covered self-expandable metallic stent (SEMS) was partially embedded in the esophagus through the tracheoesophageal fistula (TEF). **(B)** The embedded part of the stent was vaporized with the neodymium-doped yttrium aluminum garnet laser (white arrow) through the esophagus. **(C)** The covered Y-shape SEMS was removed *via* rigid bronchoscopy in a piecemeal fashion. **(D)** The bronchoscopic view of the TEF (white arrow) and complex post-tuberculosis tracheobronchial stenosis (PTTS) in the trachea.

The patient was diagnosed with stent-related TEF and complex PTTS. She underwent percutaneous endoscopic gastrostomy for enteric feeding and artificial nutrition. She was administered anti-infective therapy with cefoperazone sulbactam and acid-suppressive treatment with ranitidine. A multidisciplinary discussion was conducted among pulmonologists, gastroenterologists, cardiothoracic surgeons, and anesthesiologists. The patient consented to remove the covered Y-shape SEMS *via* bronchoscopic intervention because the surgical repair was impossible and deemed high risk. Since the stent was partially embedded in the esophagus (Figure 2A), we vaporized this embedded part with the neodymium-doped yttrium aluminum garnet laser (Figure 2B). We then gently sledded the slope of the rigid scope between the stent and the airway to avoid tearing the TEF during the stent removal. The covered Y-shape SEMS was removed *via* rigid bronchoscopy in a piecemeal fashion (Figure 2C) under general anesthesia, revealing the TEF and complex PTTS in the trachea (Figure 2D). The whole procedure was completed in concert with cardiothoracic surgery backup.

**FIGURE 3:**
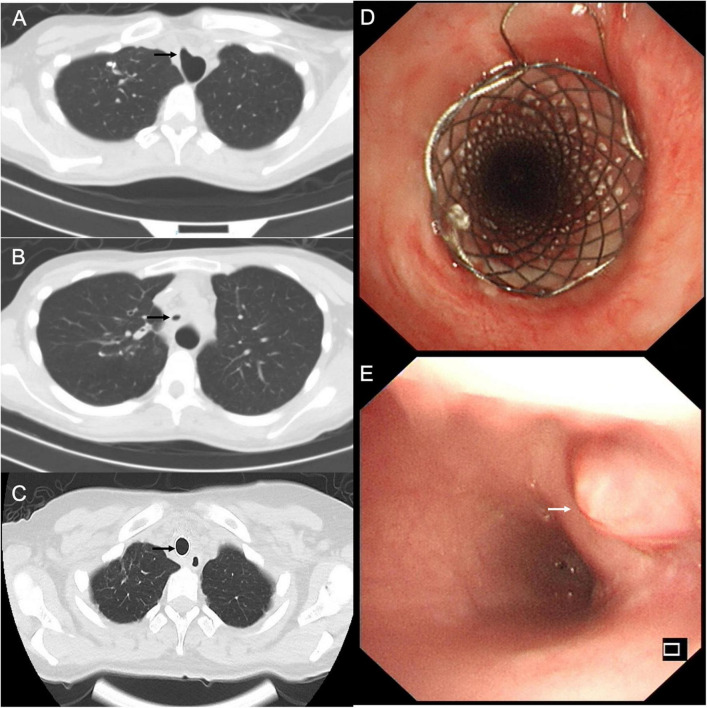
**(A)** The chest computed tomography (CT) showed the tracheoesophageal fistula (TEF) in the trachea (black arrow). **(B)** The chest CT performed after the stent removal demonstrated a diffuse tracheal stenosis, the narrowest part being 5.7 mm in diameter (black arrow). **(C)** Axial chest CT view confirmed the closure of TEF and patency of the trachea (black arrow). **(D)** The fully covered self-expandable metallic stent extended more than 10 mm beyond the TEF to occlude the orifice and establish the patency of the tracheobronchial tree. **(E)** The endoscopic view of TEF was fully covered by epithelium and granulation tissue (white arrow).

The patient presented with worsening dyspnea and severe stridor after removing the covered Y-shape SEMS. She had a large TEF (Figure 3A), associated with complex PTTS, with the narrowest part being 5.7 mm in diameter (Figure 3B), unsuitable for surgical repair or rigid bronchoscopy (which could increase the risk of TEF perforation). The patient consented to receive the insertion of another fully covered Y-shape SEMS (Microtech Co., Ltd., Nanjing, China) to occlude the TEF orifice and establish the patency of the tracheobronchial tree (Figure 3C) on a compassionate basis. The upper edge of the stent extended more than 10 mm beyond the range of the TEF (Figure 3D). The local administration of recombinant bovine basic fibroblast growth factor (rbFGF) (Beifuji Spray, Essex Bio-Pharmaceutical Co., Ltd.; 6,000 IU/time) was sprayed into and around the fistula through the esophagus using the bronchoscope every other week as we reported (for a total of 4 times) (12). The TEF was closed 3 months after inserting the fully covered Y-shaped SEMS and covered by epithelium and granulation tissue (Figure 3E) in endoscopy. The gastrostomy tube was removed when the patient started with normal feeding. The patient received routine bronchoscopy and removal of granulation tissue on the edges of the SEMS every 2-weeks to maintain the stent patency and reduce the risk of stent-related TEF. She showed sustained clinical and radiographic improvement in the follow-up up to 1.5 years after the treatment.

## Discussion

We reported an uncommon but clinically severe case of SEMS-related TEF in a patient with complex PTTS. PTTS is the most common type of tracheobronchial stenosis with uniform thickening in long bronchial segments in patients with pulmonary TB (13–15). Various bronchoscopic treatments have been applied in managing PTTB, including laser, balloon dilation, silicone stent, and fully covered SEMS (4, 16). Complex PTTS with cartilage destruction or malacia (unsuitable for surgical repair) necessitates the silicone or metallic stent insertion considering the re-obstruction rate using laser or balloon dilation alone (17). The silicone stent is more preferred than the fully covered SEMS in patients with benign airway disease after thoroughly exploring all the other treatment strategies in the advisory by the U.S. Food and Drug Administration (18). The primary technical disadvantage of silicone stent is the difficulty of inserting the rigid bronchoscopy into the distal end of the twisted and stenotic tracheobronchial tree in this case of complex PTTS (5, 19). In addition, this procedure could increase the risk of TEF perforation. Moreover, the silicone stent might not completely occlude the fistula due to its poor conformability. The patient finally consented to receive the insertion of another fully covered Y-shape SEMS on a compassionate basis, which was considered the most promising and least risky therapeutic strategy.

Although there is no randomized controlled trial of fully covered SEMS vs. silicone stent for complex PTTS, the fully covered SEMS has been reported in case reports, small studies, and expert consensuses (3–5, 9). We summarized the main results from previous studies reporting the application of fully covered SEMS in benign airway stenosis in Table 1. These studies came to controversial conclusions: some authors considered fully covered SEMS too risky (4, 20, 21), while others thought them as a safe and effective technique for benign airway stenosis, but should be closely monitored (3, 22–25). The popularity of fully covered SEMS has sparked concerns about stent-related complications, including granulation tissue formation, stent migration, mucus retention, bacterial overgrowth, pneumonia et al. (3–5, 9). Granulation tissue forms at both edges of the stent, resulting in airway obstruction (3–5, 9). Stent removal in benign airway disease is also associated with complications such as mucosal tears, severe bleeding, re-obstruction, and respiratory failure, resulting in longer hospital stays and higher medical costs (18). While the rates of complications requiring stent removal were significant as demonstrated in Table 1, no life-threatening complications occurred in patients with benign airway stenosis receiving the fully covered SEMS, which are not easily embedded into the mucosal wall. In this case, the severe granulation tissue hyperplasia on the upper edge of SEMS might increase the shearing force of the stent against the anterior esophageal wall and the subsequent friction leading to the formation of TEF as indicated in other studies (26, 27). The stent-related TEF does not heal spontaneously, thus posing a major dilemma in managing complex PTTS, for which surgical repair is impossible and deemed high risk. The SEMS was removed in three steps: (1) vaporizing the embedded part of the stent in the esophagus, (2) sliding the slope of the rigid scope gently between the stent and the airway, (3) extracting the stent as a whole or in a piecemeal fashion when necessary.

**TABLE 1:** Previously reported studies on fully covered SEMS in benign airway stenosis.

	Year	Authors	Patients	SEMS metal	SEMS cover	Follow-up (days)	Overall complication rate	Most frequent complications	Requiring stent removing	Stent-remove complications
1	2006	Shin et al. (23)	6	Nitinol	PTFE	270 (range, 30–550)	83% (5/6)	Granulation (50%), migration (40%)	67% (4/6)	NM
2	2009	Fernandez-Bussy et al. (35)	24	Nitinol	Silicone	232 (range, 40–424)	49% (24/49)	Granulation (20.4%), migration (18.4%)	49% (24/49)	NM
3	2009	Dooms et al. (20)	17	Nitinol	Silicone, PTFE, PU	56 (range, 1–392)	90% (18/20)	Migration (60%), wrinkling (10%)	80% (16/20)	NM
4	2014	Chen et al. (22)	21	Stainless steel	Silastic	750 (range, 30–1080)	91% (19/21)	Granulation (85.7%), migration (9.5%)	19% (4/21)	NM
5	2016	Dahlqvist et al. (24)	16	Nitinol	Silicone	282 (range, 0–1618)	75% (15/21)	Granulation (33.3%), migration (28.6%)	55% (11/21)	No mucosal tear or bleeding
6	2017	Fortin et al. (3)	30	Nitinol	Silicone	224 ± 96	50% (20/40)	Migration (32.5%), granulation (7.5%),	50% (20/40)	NM
7	2017	Han et al. (25)	21	Nitinol	Silicone	60 (rang, 29–103)	100% (21/21)	Granulation (47.6%)	50% (10/21)	No mucosal tear or bleeding
8	2019	Xiong et al. (4)	32	Nitinol	Silicone	32 (2–142)	32% (19/59)	Granulation (15.2%)	32% (19/59)	NM
9	2020	Jeong et al. (21)	19	NM	NM	90 (60–240)	100% (19/19)	Granulation (100%), migration (20%)	100% (19/19)	Bleeding (10%), fistula (5%)

SEMS, self-expandable metallic stents; PTFE, polytetrafluoroethylene; PU, polyurethane; NM, not mentioned.

In their review of airway stenting, Prof. Guibert and his colleagues highlighted that fully covered SEMS are partially suitable for tracheoesophageal fistulas, tight and deformed stenoses, and more generally for extremely necrotic stenoses, eliminating the need to insert the rigid bronchoscope across the stenosis (which could increase the risk of TEF perforation) (28). The decision to insert another covered SEMS following the removal was to occlude the TEF and restore the patency in the tracheobronchial tree. The fully covered SEMS is still controversial in the management of benign airway disease for its associated complications and difficulty in endobronchial removal (18). This strategy must be weighed with the stent-related long-term complications in a proper and timely way. The routine bronchoscopy and removal of granulation tissue on the edges of covered SEMS are necessary to maintain the stent patency and reduce the risk of stent-related TEF in the follow-up. The fully covered SEMS could be a bridge to silicone stent placement, increasing the success rate especially in severe PTTB patients (5).

Fistula healing is a complex process involving granulation tissue formation, epithelialization, extracellular matrix formation, and wound remodeling (29). rbFGF has been reported to close the bronchopleural fistula by prompting epithelialization, vascularization, and wound remodeling (12, 30–32). The air leak through the fistula is associated with high mortality and bronchoscopic treatment failure in the management of bronchopleural fistula (33, 34). The bronchoscopic spray of rbFGF *via* the esophagus could be a complementary procedure to the covered SEMS (reducing air leak and increasing the topical rbFGF concentration) for the closure of TEF.

## Conclusion

We presented the bronchoscopic management of a stent-related TEF in a patient with complex PTTS. The stent was removed in three steps. The fully covered SEMS seems to be an alternative and one-stage procedure for the temporary management of TEF combined with complex PTTS. The bronchoscopic spray of rbFGF could be a complementary procedure to the covered SEMS. We recommend biweekly mechanical and thermal removal of granulation tissue at the edges of the covered SEMS to maintain the stent patency and reduce the risk of stent-related TEF. More studies are needed to validate the safety and effectiveness of the novel SEMS in the context of benign airway disease.

## Data availability statement

The original contributions presented in this study are included in the article/supplementary material, further inquiries can be directed to the corresponding authors.

## Ethics statement

The studies involving human participants were reviewed and approved by the Institutional Review Boards of The First Affiliated Hospital of Chongqing Medical University. The patients/participants provided their written informed consent to participate in this study.

## Author contributions

SG and YL were the chief pulmonary intervention physicians. YB, YY, JC, and SL wrote the manuscript and were involved in editing the manuscript. All authors contributed to the article and approved the submitted version.
